# Supplementary material for: Genetic Contribution of Endometriosis to the Risk of Developing Hormone-Related Cancers
Source: Int J Mol Sci. 2021 Jun 4;22(11):6083. doi: 10.3390/ijms22116083 (PMC8200110; doi:10.3390/ijms22116083)
Supplement: Supplementary file 1 [file ijms-22-06083-s001.zip › ijms-1244314-supplementary/Rueda-et-al_IJMS_2021-06-04_SUPPLEMENTARY-FIGURES-TABLES/Rueda-et-al_IJMS_2021-05-17_Supplementary-file-1_R-scripts.pdf]

```
### Iraia Garcia-Santisteban, 2021
### 2SMR analysis using public data
### Exposure: endometriosis; Outcomes: hormone-related cancers (ovarian,
breast, endometrial cancers)

# Set working directory (the path will be specific to your directory):
setwd("/home/iraia/2SMR_Endometriosis/")

# Load libraries:
library(TwoSampleMR)
library(MRInstruments)
library(ggplot2)

######################################################################

# Global 2SMR (IVW, WM, MRE), Single SNP 2SMR (Wald ratio) with forest
plots & Sensitivity analyses (heterogeneity, horizontal pleiotropy)
# Exposure: endometriosis, Sapkota et al. 2017, European ancestry women
(DISCOVERY)
# Outcomes: hormone-related cancers (ovarian, breast, endometrial)

# Prepare exposure data (endometriosis)
gwas_Sapkota <-subset(gwas_catalog,grepl("GCST004549",STUDY.ACCESSION))
gwas_Sapkota_EA <-subset(gwas_Sapkota,grepl("(EA)",Phenotype_info))
exp_gine <- format_data(gwas_Sapkota_EA)
exp_dat <- clump_data(exp_gine)
rm(gwas_catalog,gwas_Sapkota,exp_gine,gwas_Sapkota_EA)

# Prepare outcome data (hormone-related cancers cancers)
# Ovarian cancer:
# Phelan 2017 NatGen, PMID 28346442
out_dat1.1  <-  extract_outcome_data(snps=exp_dat$SNP,  outcomes='ieu-a-
1120') # Ovarian cancer

# Breast cancer:
# Michailidou 2017 Nature, PMID 29059683
out_dat2.1  <-  extract_outcome_data(snps=exp_dat$SNP,  outcomes='ieu-a-
1126') # Breast cancer

# Endometrial cancer:
# O'Mara 2018 NatComm, PMID 30093612
out_dat3.1 <- read_outcome_data(
  snps = exp_dat$SNP,
  filename = "30093612-GCST006464-EFO_1001512-Build37.tsv",
  sep = "\t",
  snp_col = "variant_id",
  beta_col = "beta",
  se_col = "standard_error",
  effect_allele_col = "effect_allele",
  other_allele_col = "other_allele",
  eaf_col = "effect_allele_frequency",
  pval_col = "p_value",
  units_col = "",
  gene_col = "",
  samplesize_col = "")

# Harmonise exposure and outcome data
dat1.1 <- harmonise_data(exp_dat, out_dat1.1)
dat2.1 <- harmonise_data(exp_dat, out_dat2.1)
dat3.1 <- harmonise_data(exp_dat, out_dat3.1)
```

```
# Perform overall 2SMR analysis
res1.1_mr     <-     mr(dat1.1,     method_list=c("mr_weighted_median", "mr_egger_regression", "mr_ivw"))
res2.1_mr     <-     mr(dat2.1,     method_list=c("mr_weighted_median", "mr_egger_regression", "mr_ivw"))
res3.1_mr     <-     mr(dat3.1,     method_list=c("mr_weighted_median", "mr_egger_regression", "mr_ivw"))

# Perform single SNP 2SMR analysis
res1.1_single <- mr_singlesnp(dat1.1, all_method="mr_ivw")
res2.1_single <- mr_singlesnp(dat2.1, all_method="mr_ivw")
res3.1_single <- mr_singlesnp(dat3.1, all_method="mr_ivw")

# Make forest plots:
FP1.1 <- mr_forest_plot(res1.1_single)
FP1.1[[1]]
FP2.1 <- mr_forest_plot(res2.1_single)
FP2.1[[1]]
FP2.3 <- mr_forest_plot(res2.3_single)
FP2.3[[1]]
FP3.1 <- mr_forest_plot(res3.1_single)
FP3.1[[1]]

# Perform heterogeneity tests:
het1.1 <- mr_heterogeneity(dat1.1)
het2.1 <- mr_heterogeneity(dat2.1)
het3.1 <- mr_heterogeneity(dat3.1)

# Perform horizontal pleiotropy tests:
plei1.1 <- mr_pleiotropy_test(dat1.1)
plei2.1 <- mr_pleiotropy_test(dat2.1)
plei3.1 <- mr_pleiotropy_test(dat3.1)

########################################################################

# Global 2SMR (IVW, WM, MRE)
# Exposure: endometriosis, UKBB cohort data (REPLICATION)
# Outcomes: hormone-related cancers (ovarian, breast, endometrial)

# Prepare exposure data (UKBB Non-cancer illness code, self-reported: endometriosis)
exp_dat <- extract_instruments("ukb-b-10903")

# Prepare outcome data (hormone-related cancers cancers)
# Ovarian cancer:
# Phelan 2017 NatGen, PMID 28346442
out_dat1.1  <-  extract_outcome_data(snps=exp_dat$SNP,  outcomes='ieu-a-1120') # Ovarian cancer

# Breast cancer:
# Michailidou 2017 Nature, PMID 29059683
out_dat2.1  <-  extract_outcome_data(snps=exp_dat$SNP,  outcomes='ieu-a-1126') # Breast cancer

# Endometrial cancer:
# O’Mara 2018 NatComm, PMID 30093612
out_dat3.1 <- read_outcome_data(
  snps = exp_dat$SNP,
  filename = "30093612-GCST006464-EFO_1001512-Build37.tsv",
  sep = "\t",
  snp_col = "variant_id",
```

```
  beta_col = "beta",
  se_col = "standard_error",
  effect_allele_col = "effect_allele",
  other_allele_col = "other_allele",
  eaf_col = "effect_allele_frequency",
  pval_col = "p_value",
  units_col = "",
  gene_col = "",
  samplesize_col = "")

# Harmonise exposure and outcome data
dat1.1 <- harmonise_data(exp_dat, out_dat1.1)
dat2.1 <- harmonise_data(exp_dat, out_dat2.1)
dat3.1 <- harmonise_data(exp_dat, out_dat3.1)

# Perform overall 2SMR analysis
res1.1_mr     <-     mr(dat1.1,     method_list=c("mr_weighted_median",
"mr_egger_regression", "mr_ivw"))
res2.1_mr     <-     mr(dat2.1,     method_list=c("mr_weighted_median",
"mr_egger_regression", "mr_ivw"))
res3.1_mr     <-     mr(dat3.1,     method_list=c("mr_weighted_median",
"mr_egger_regression", "mr_ivw"))

######################################################################
# Global 2SMR (IVW) & Scatter plots
# Exposure: endometriosis, Sapkota et al. 2017, European ancestry women
# Outcomes: ovarian cancer histotypes

#  Prepare  exposure  data  (Endometriosis,  Sapkota,  European  Ancestry
women)
gwas_Sapkota <-subset(gwas_catalog,grepl("GCST004549",STUDY.ACCESSION))
gwas_Sapkota_EA <-subset(gwas_Sapkota,grepl("(EA)",Phenotype_info))
exp_gine <- format_data(gwas_Sapkota_EA)
exp_dat <- clump_data(exp_gine)
rm(gwas_Sapkota,exp_gine,gwas_Sapkota_EA)

# Prepare outcome data (ovarian cancer histotypes)
# Phelan 2017 NatGen, PMID 28346442
# Ovarian cancer (all available histotypes):
# ieu-a-1120: Ovarian cancer
# ieu-a-1124: Clear cell o. c.
# ieu-a-1125: Endometroid o. c.
# ieu-a-1121: High grade serous o. c.
# ieu-a-1122: Low grade serous o. c.
# ieu-a-1123: Invasive mucinous o. c.
# ieu-a-1228: High and low grade serous o. c.
# ieu-a-1229: Serous o. c.: low grade, low malignant potential
# ieu-a-1230: Serous o. c.: low malignant potential
# ieu-a-1231: Mucinous o. c.: invasive, low malignant potential
# ieu-a-1232: Low malignant potential mucinous o. c.
# ieu-a-1233: Low malignant potential o. c.

out_dat <-extract_outcome_data(snps=exp_dat$SNP,
                               outcomes=c(
                                 "ieu-a-1120",
                                 "ieu-a-1124",
                                 "ieu-a-1125",
                                 "ieu-a-1121",
                                 "ieu-a-1122",
                                 "ieu-a-1123",
                                 "ieu-a-1228",
```

```
                                  "ieu-a-1229",
                                  "ieu-a-1230",
                                  "ieu-a-1231",
                                  "ieu-a-1232",
                                  "ieu-a-1233"))

# Harmonise exposure and outcome data
dat <- harmonise_data(exp_dat, out_dat)

# Perform 2SMR analysis (MR); only IVW method.
res_mr <- mr(dat, method="mr_ivw")

# Make scatter plots:
SP <- mr_scatter_plot(res_mr, dat)
SP[[1]]
SP[[2]]
SP[[3]]
SP[[4]]
SP[[5]]
SP[[6]]
SP[[7]]
SP[[8]]
SP[[9]]
SP[[10]]
SP[[11]]
SP[[12]]
```
