# Supplementary material for: Genetic Contribution of Endometriosis to the Risk of Developing Hormone-Related Cancers
Source: Int J Mol Sci. 2021 Jun 4;22(11):6083. doi: 10.3390/ijms22116083 (PMC8200110; doi:10.3390/ijms22116083)
Supplement: Supplementary file 1 [file ijms-22-06083-s001.zip › ijms-1244314-supplementary/Rueda-et-al_IJMS_2021-06-04_SUPPLEMENTARY-FIGURES-TABLES/Rueda-et-al_IJMS_2021-06-04_Supplementary-figures-tables.pptx]

## Slide 1
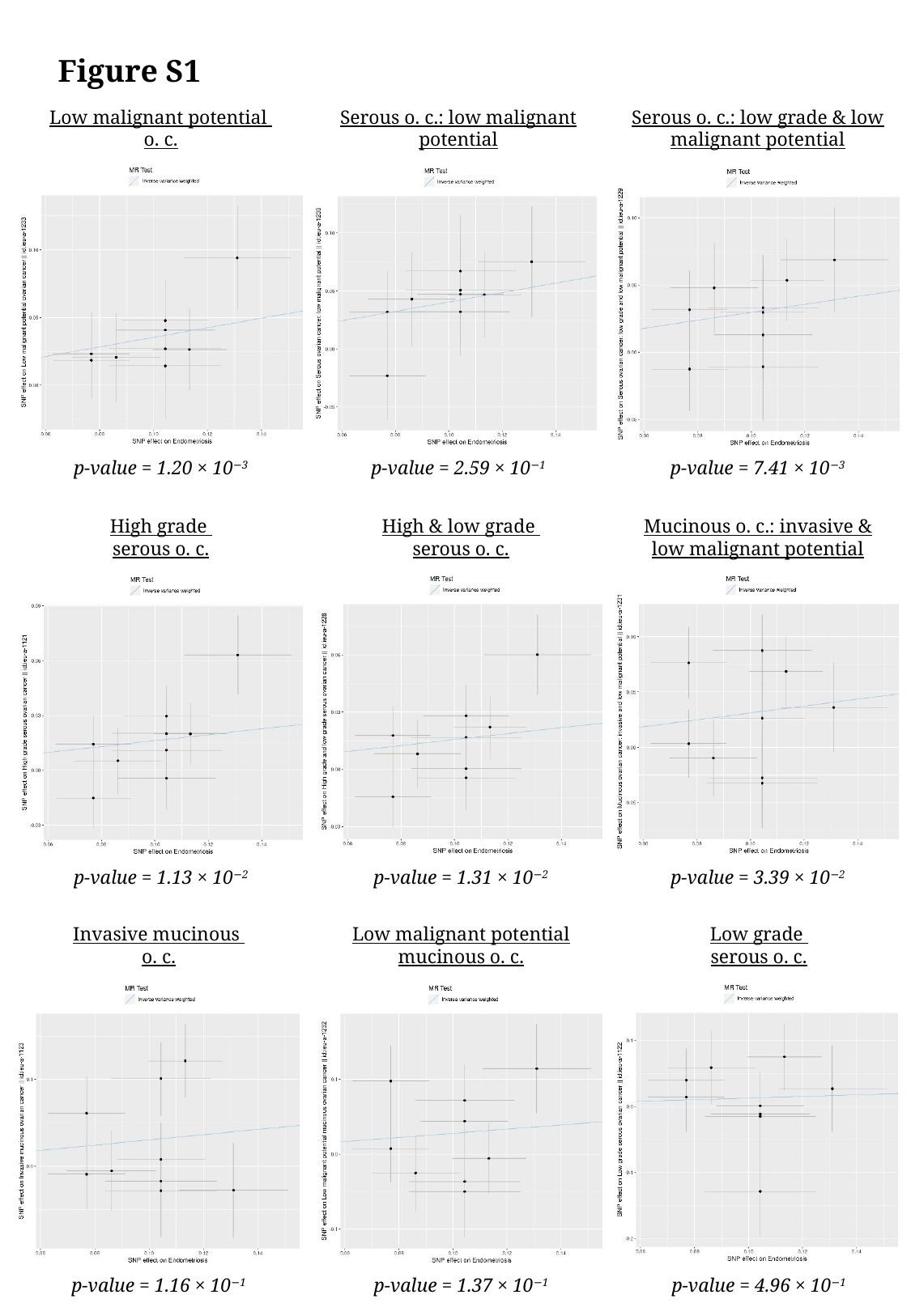

Figure S1
Low malignant potential
o. c.
p-value = 1.20 × 10^−3^
Serous o. c.: low malignant potential
p-value = 2.59 × 10^−1^
Serous o. c.: low grade & low malignant potential
p-value = 7.41 × 10^−3^
High grade
serous o. c.
p-value = 1.13 × 10^−2^
High & low grade
serous o. c.
p-value = 1.31 × 10^−2^
Mucinous o. c.: invasive & low malignant potential
p-value = 3.39 × 10^−2^
Invasive mucinous
o. c.
p-value = 1.16 × 10^−1^
Low malignant potential mucinous o. c.
p-value = 1.37 × 10^−1^
Low grade
serous o. c.
p-value = 4.96 × 10^−1^

## Slide 2
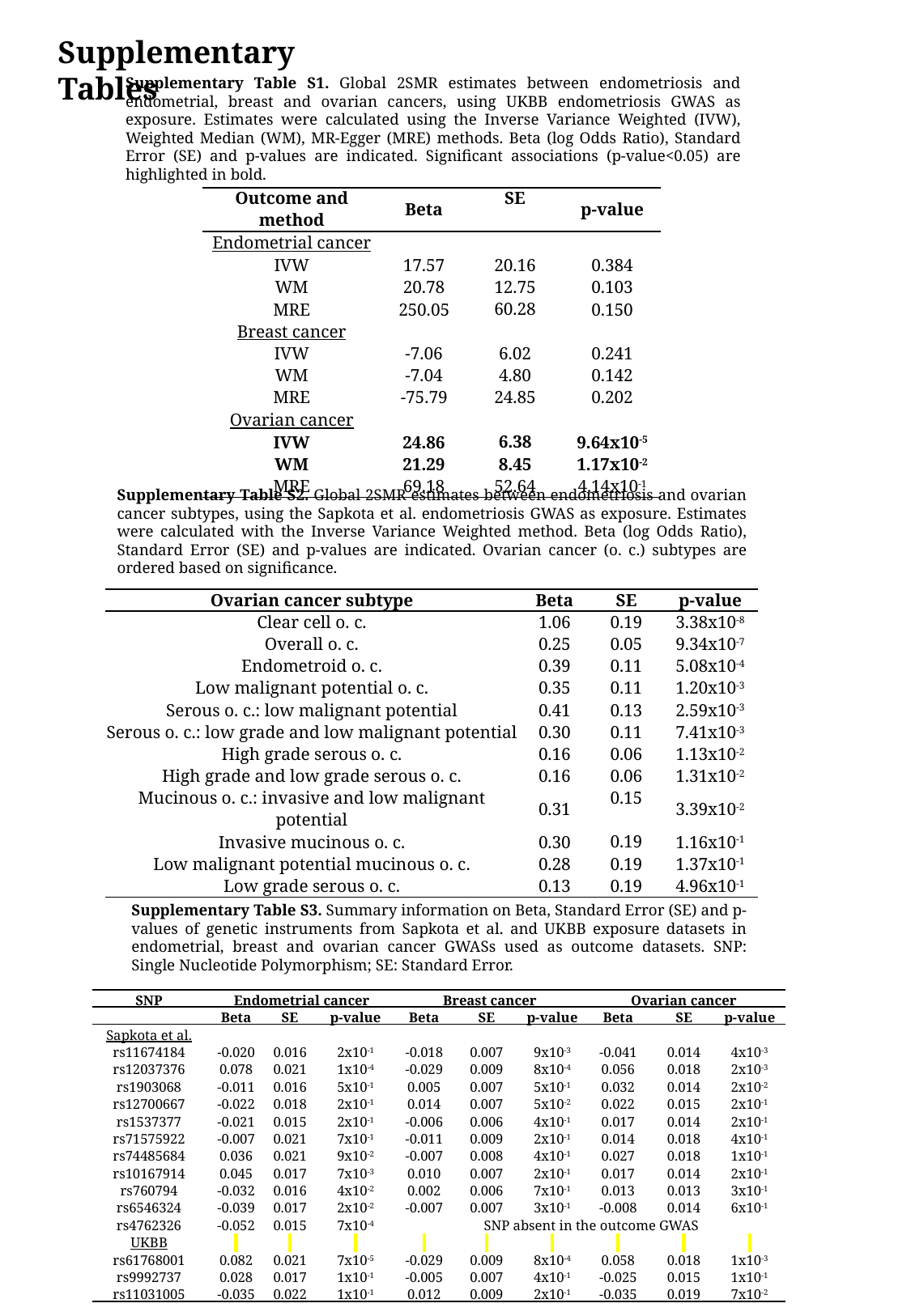

Supplementary Tables
Supplementary Table S1. Global 2SMR estimates between endometriosis and endometrial, breast and ovarian cancers, using UKBB endometriosis GWAS as exposure. Estimates were calculated using the Inverse Variance Weighted (IVW), Weighted Median (WM), MR-Egger (MRE) methods. Beta (log Odds Ratio), Standard Error (SE) and p-values are indicated. Significant associations (p-value<0.05) are highlighted in bold.
| Outcome and method | Beta | SE | p-value | |
| --- | --- | --- | --- | --- |
| Endometrial cancer | | | | |
| IVW | 17.57 | 20.16 | 0.384 | |
| WM | 20.78 | 12.75 | 0.103 | |
| MRE | 250.05 | 60.28 | 0.150 | |
| Breast cancer | | | | |
| IVW | -7.06 | 6.02 | 0.241 | |
| WM | -7.04 | 4.80 | 0.142 | |
| MRE | -75.79 | 24.85 | 0.202 | |
| Ovarian cancer | | | | |
| IVW | 24.86 | 6.38 | 9.64x10^-5^ | |
| WM | 21.29 | 8.45 | 1.17x10^-2^ | |
| MRE | 69.18 | 52.64 | 4.14x10^-1^ | |
Supplementary Table S2. Global 2SMR estimates between endometriosis and ovarian cancer subtypes, using the Sapkota et al. endometriosis GWAS as exposure. Estimates were calculated with the Inverse Variance Weighted method. Beta (log Odds Ratio), Standard Error (SE) and p-values are indicated. Ovarian cancer (o. c.) subtypes are ordered based on significance.
| Ovarian cancer subtype | Beta | SE | p-value |
| --- | --- | --- | --- |
| Clear cell o. c. | 1.06 | 0.19 | 3.38x10^-8^ |
| Overall o. c. | 0.25 | 0.05 | 9.34x10^-7^ |
| Endometroid o. c. | 0.39 | 0.11 | 5.08x10^-4^ |
| Low malignant potential o. c. | 0.35 | 0.11 | 1.20x10^-3^ |
| Serous o. c.: low malignant potential | 0.41 | 0.13 | 2.59x10^-3^ |
| Serous o. c.: low grade and low malignant potential | 0.30 | 0.11 | 7.41x10^-3^ |
| High grade serous o. c. | 0.16 | 0.06 | 1.13x10^-2^ |
| High grade and low grade serous o. c. | 0.16 | 0.06 | 1.31x10^-2^ |
| Mucinous o. c.: invasive and low malignant potential | 0.31 | 0.15 | 3.39x10^-2^ |
| Invasive mucinous o. c. | 0.30 | 0.19 | 1.16x10^-1^ |
| Low malignant potential mucinous o. c. | 0.28 | 0.19 | 1.37x10^-1^ |
| Low grade serous o. c. | 0.13 | 0.19 | 4.96x10^-1^ |
Supplementary Table S3. Summary information on Beta, Standard Error (SE) and p-values of genetic instruments from Sapkota et al. and UKBB exposure datasets in endometrial, breast and ovarian cancer GWASs used as outcome datasets. SNP: Single Nucleotide Polymorphism; SE: Standard Error.
| SNP | Endometrial cancer | | | Breast cancer | | | Ovarian cancer | | |
| --- | --- | --- | --- | --- | --- | --- | --- | --- | --- |
| | Beta | SE | p-value | Beta | SE | p-value | Beta | SE | p-value |
| Sapkota et al. | | | | | | | | | |
| rs11674184 | -0.020 | 0.016 | 2x10^-1^ | -0.018 | 0.007 | 9x10^-3^ | -0.041 | 0.014 | 4x10^-3^ |
| rs12037376 | 0.078 | 0.021 | 1x10^-4^ | -0.029 | 0.009 | 8x10^-4^ | 0.056 | 0.018 | 2x10^-3^ |
| rs1903068 | -0.011 | 0.016 | 5x10^-1^ | 0.005 | 0.007 | 5x10^-1^ | 0.032 | 0.014 | 2x10^-2^ |
| rs12700667 | -0.022 | 0.018 | 2x10^-1^ | 0.014 | 0.007 | 5x10^-2^ | 0.022 | 0.015 | 2x10^-1^ |
| rs1537377 | -0.021 | 0.015 | 2x10^-1^ | -0.006 | 0.006 | 4x10^-1^ | 0.017 | 0.014 | 2x10^-1^ |
| rs71575922 | -0.007 | 0.021 | 7x10^-1^ | -0.011 | 0.009 | 2x10^-1^ | 0.014 | 0.018 | 4x10^-1^ |
| rs74485684 | 0.036 | 0.021 | 9x10^-2^ | -0.007 | 0.008 | 4x10^-1^ | 0.027 | 0.018 | 1x10^-1^ |
| rs10167914 | 0.045 | 0.017 | 7x10^-3^ | 0.010 | 0.007 | 2x10^-1^ | 0.017 | 0.014 | 2x10^-1^ |
| rs760794 | -0.032 | 0.016 | 4x10^-2^ | 0.002 | 0.006 | 7x10^-1^ | 0.013 | 0.013 | 3x10^-1^ |
| rs6546324 | -0.039 | 0.017 | 2x10^-2^ | -0.007 | 0.007 | 3x10^-1^ | -0.008 | 0.014 | 6x10^-1^ |
| rs4762326 | -0.052 | 0.015 | 7x10^-4^ | SNP absent in the outcome GWAS | | | | | |
| UKBB | | | | | | | | | |
| rs61768001 | 0.082 | 0.021 | 7x10^-5^ | -0.029 | 0.009 | 8x10^-4^ | 0.058 | 0.018 | 1x10^-3^ |
| rs9992737 | 0.028 | 0.017 | 1x10^-1^ | -0.005 | 0.007 | 4x10^-1^ | -0.025 | 0.015 | 1x10^-1^ |
| rs11031005 | -0.035 | 0.022 | 1x10^-1^ | 0.012 | 0.009 | 2x10^-1^ | -0.035 | 0.019 | 7x10^-2^ |
